# Seven years of use of implantable cardioverter-defibrillator therapies: a nationwide population-based assessment of their effectiveness in real clinical settings

**DOI:** 10.1186/s12872-015-0016-2

**Published:** 2015-03-13

**Authors:** Arn Migowski, Antonio Luiz Ribeiro, Marilia Sá Carvalho, Vitor Manuel Pereira Azevedo, Rogério Brant Martins Chaves, Lucas de Aquino Hashimoto, Carolina de Aquino Xavier, Regina Maria de Aquino Xavier

**Affiliations:** Instituto Nacional de Cardiologia - INC (National Institute of Cardiology, Ministry of Health), Coordenação de Ensino e Pesquisa, Divisão de Saúde Coletiva, rua das Laranjeiras 374, Laranjeiras, Rio de Janeiro, RJ Brazil; University Hospital and School of Medicine, Federal University of Minas Gerais (UFMG), Minas Gerais, Brazil; Oswaldo Cruz Foundation (FIOCRUZ), Rio de Janeiro, Brazil; School of Medicine, Federal University of Rio de Janeiro (UFRJ), Rio de Janeiro, Brazil

**Keywords:** Implantable defibrillators, Cardiac resynchronization therapy devices, Chagas cardiomyopathy, Survival analysis, Medical record linkage, Brazil, Database, Technology assessment, Hospital mortality, High-cost technology

## Abstract

**Background:**

The efficacy of implantable cardioverter-defibrillator (ICD) and cardiac resynchronization therapy-defibrillator (CRT-D) therapy has already been established in clinical trials but their effectiveness in several clinical settings remains undetermined. This study aimed to assess the effectiveness of ICD and CRT-D therapies within the Brazilian National Health System (SUS).

**Methods:**

All patients who underwent ICD or CRT-D implantation within the SUS from 2001 to 2007 were included in the study. We compared estimated Kaplan-Meier survival curves using the Peto’s test. Prognostic factors were selected using Cox’s models.

**Results:**

There were included 3,295 patients in the ICD group and 681 patients in the CRT-D group. Cardiac causes accounted for 79% of all deaths in both groups and Chagas’ heart disease accounted for 31% of these deaths. In the CRT-D group, survival significantly decreased around the fourth year of follow-up, with a decrease from 59.5% to 38.3% in 5.5 months. Transvenous implantation technique was used in 62% of CRT-D patients. In-hospital case-fatality rates were higher in those undergoing surgical implantation (5.3%) than those undergoing transvenous implantation (1.6%) (p = 0.02).

**Conclusions:**

The results show that short-term, medium-term and long-term effectiveness of ICD therapy appears to be similar to that evidenced in clinical trials. In the CRT-D group, in-hospital case-fatality and 30-day case-fatality were higher than those reported in other studies. Surgical epicardial implantation technique was performed in this group at a higher frequency than that reported in the literature and was associated with poorer short-term prognosis.

## Background

The efficacy of implantable cardioverter-defibrillator (ICD) therapy for primary and secondary prevention of sudden cardiac death has been established in several clinical scenarios in patients with both ischemic and nonischemic heart disease [1]. The efficacy of cardiac resynchronization therapy combined with ICD (cardiac resynchronization therapy-defibrillator, CRT-D) in reducing overall mortality has also been shown in some clinical settings compared with optimal pharmacological therapy [2], ICD alone [3] or even with the CRT-alone [4].

However, clinical trials do not assess the effectiveness of these therapies under real life conditions of use where patient follow-up is a common problem [5]. Other common issues include inappropriate indications, suboptimal adherence to guidelines and off-label uses [6], more heterogeneous patient populations, and potential provider- or device-related technical shortcomings. Furthermore, evidence showing long-term effectiveness of these therapies is scarce [7] and some controversial issues have not been properly addressed in clinical trials, including indications of dual-chamber ICD [8], use of ICD and CRT-D in children and adolescents [9] and in patients with Chagas’ heart disease [10].

In the light of these issues and scarcity of population-based data on ICD implantation [11] particularly lack of national registries—administrative databases have increasingly gained importance as a source of information complementary to data obtained from clinical trials and specific records [12].

Therefore, probabilistic record linkage techniques have been used in cardiology research to analyze population data from routine hospital administrative databases and nationwide death records [13]. These national databases are essential sources of information in countries with great population heterogeneity and a wide range of patterns of therapy utilization.

The present study aimed to assess short-term, medium-term and long-term survival of ICD and CRT-D therapies within the Brazilian National Health System (SUS) using record linkage between two national databases.

## Methods

### Study population and data sources

Two national databases were used as data sources for the study: the Brazilian Mortality Database (known by its Portuguese acronym SIM) and the Brazilian Hospital Information Database (known by its Portuguese acronym SIH). SIM was created in 1975 and covers the entire population nationwide. Mortality data is considered reliable from the qualitative point of view, as accurate as that of other countries with a long tradition in these statistics [14]. SIH was created in 1981 and covers the entire Brazilian National Health System (SUS), which provides universal health coverage for over 200 million people, with 75% of them covered exclusively by it. The accuracy of the SIH variables related to the diagnosis, medical procedures, sex, age-group and in-hospital outcomes are considered satisfactory [15].

Our study cohort consisted of all patients admitted to SUS hospitals (either public or SUS-affiliated private) undergoing transvenous ICD or CRT-D implantation from 2001 to 2007. The main clinical indications (Class I) for ICD implantation within SUS during the study period following the Brazilian Ministry of Health guidelines included: cardiac arrest due to ventricular tachycardia or ventricular fibrillation from irreversible causes in patients with EF ≤35%; spontaneous sustained ventricular tachycardia from irreversible causes in patients with EF ≤35%; non-sustained ventricular tachycardia with previous acute myocardial infarction, left ventricular dysfunction (EF ≤40%) and sustained ventricular tachycardia or ventricular fibrillation inducible at programmed ventricular stimulation. CRT-D therapy was primarily indicated for patients meeting one of the above criteria for ICD implantation and QRS duration equal to or greater than 130 ms, functional class III or IV (The New York Heart Association [NYHA] Functional Classification) and left ventricle end-diastolic diameter equal to or greater than 55 mm and EF ≤0.30.

A probabilistic record linkage technique was used to find death records for each patient in the national SIM database during the study period. We chose to apply the probabilistic record linkage as there is no unique identifier between SIM and SIH databases. The linkage method applied showed 90.6% sensitivity and 100% specificity [16].

### Data analysis

We performed an overall survival and cardiac survival analysis considering only deaths from any underlying cardiac cause (including Chagas’ heart disease and congenital heart disease), procedure-related complications or other causes potentially related to sudden cardiac death according to the following codes: T821; I00-I528; B570-B572; Q200-Q249; R570; R960, and R98 (International Statistical Classification of Diseases and Related Health Problems, 10th Revision).

Patients who did not die by the end of the study period (12/31/2007) were censored. No patient was lost to follow-up during the study period assuming universal coverage of SIM nationwide and no deaths occurring abroad. As for cardiac survival rates, patients whose underlying cause of death was not defined as of cardiac origin were also censored and were included in the analysis on the date of death or on the last date of observation. The start time of observation for each individual (T_0_) was the date of hospitalization for the implantation procedure. If a patient underwent more than one ICD or CRT-D implantation, only the first procedure was analyzed. Generator replacement and/or lead revision procedures or any other procedures not related to device implantation were disregarded.

Due to differences in clinical eligibility criteria, patients were divided into two therapy groups for the analysis: ICD alone and CRT-D. Survival curves were estimated using the Kaplan-Meier method and compared using Peto’s test at a significance level of p <0.05. The variables selected in the univariate Cox proportional hazards regression models—adjusted for age in years—were included in the multivariate models to estimate the independent effect of the variables. The following variables were studied in the models in both groups: age; gender; hospital category; year of device implantation; and hospital location (state). We analyzed type of device (dual- or single-chamber) in the ICD-alone group and implantation technique (transvenous or mini-thoracotomy) in the CRT-D group. Separate models were constructed for overall and cardiac survival analysis stratified by therapy group (ICD alone or CRT-D). We estimated hazard ratios (HR) and their related 95% confidence intervals. Schoenfeld residuals were used to test the proportional hazards assumption of Cox models.

In Brazil, hospital admissions authorization (AIH) forms include a field for the principal diagnosis of current admission and a second one for secondary diagnosis. In the ICD-alone group, 91.83% of admission forms included the arrhythmia code as principal diagnosis but did not provide any information on the underlying disease. In the CRT-D group, a greater proportion of patient forms (33.3%) included information on the underlying disease at admission. Since there was no information on the underlying disease for many patients, we thus chose not to include this variable in the Cox models. We therefore included a new variable for underlying cardiac disease using all the disease codes from the two AIH fields and the five SIM fields, including the “contributing cause”, which may also display several codes for diseases not directly associated with the death. As a result, we were able to determine underlying disease for another 581 patients.

In-hospital case fatality was estimated based on deaths occurring during the admission when the first ICD or CRT-D implantation was performed. We calculated mean length of stay of these admissions.

For the comparison of means, we performed Student’s t-test for variables with normal distribution or otherwise the Mann-Whitney U-test. For the comparison of proportions, we used the chi-squared test. The statistical significance was set at p < 0.05. Data analyses were performed using the R Statistical Package, version 2.6.2.

This study was approved by the research ethics committee (name: Comitê de Ética em Pesquisa COEP-UFMG, protocol number 0084.0.203.000-09) and followed the principles of the Declaration of Helsinki. The ethics committee waived the requirement for written informed consent due to the study’s design.

## Results

The ICD group comprised 3,295 patients from 85 hospitals with a mean observation time of about 2.5 years, maximum follow-up time of approximately 7 years and 799 deaths were observed. CRT-D therapy became available to SUS patients in 2002, and 681 patients from 50 hospitals had received the device by 2007. The mean follow-up time in the CRT-D group was 16 months, maximum follow-up time was slightly over 5 years and 197 deaths were observed.

Single-chamber ICDs were more often implanted (64%) than dual-chamber devices. Dual-chamber devices became available to SUS patients in 2004 and accounted for 65% of all devices implanted in the ICD-alone group during 2005–2007. In general, the variables studied were similar in patients receiving single-chamber ICDs, dual-chamber ICDs and CRT-D (Table 1). The mean age was lower in the ICD-alone than CRT-D group (58 vs. 61 years, p < 0.001). The CRT-D group had a higher proportion of elderly (70 years or older) (p <0.01) and lower proportion of patients aged 10 to 49 years (p <0.001) when compared to the ICD-alone group. A comparison of device implant between Brazilian states showed that most procedures—especially CRT-D—were performed in care facilities in São Paulo, which is the richest and most industrialized state in Brazil. We found a lower proportion of supraventricular tachycardia among patients receiving dual-chamber compared to single-chamber ICDs (p <0.001).

**Table 1:** **Baseline patient characteristics stratified by type of ICD**

		**Type of ICD**					
**Characteristics**		**Single-chamber ICD**		**Dual-chamber ICD**		**CRT-D**	
		**(n = 2,109)**		**(n = 1,186)**		**(n = 681)**	
Age (years), mean (SD)		56	(±14)	57	(±14)	60	(±12)
Age group, n (%)							
	<10 years	6	(0.3)	3	(0.3)	2	(0.3)
	10 to 49 years	580	(27.5)	321	(27.1)	127	(18.7)
	50 to 59 years	580	(27.5)	310	(26.1)	189	(27.8)
	60 to 69 years	587	(27.8)	362	(30.5)	219	(32.2)
	70 years or more	356	(16.9)	190	(16.0)	143	(21.0)
Sex, n (%)							
	Female	626	(29.7)	365	(30.8)	176	(25.8)
	Male	1482	(70.3)	821	(69.2)	505	(74.2)
Arrhythmia, n (%)							
	Ventricular Flutter or Fibrillation	407	(19.3)	356	(30.0)	13	(1.9)
	Ventricular Tachycardia	914	(43.3)	618	(52.1)	242	(35.5)
	Supraventricular Tachycardia	514	(24.4)	101	(8.5)	2	(0.3)
Hospital location (state), n (%)							
	São Paulo	1069	(50.7)	703	(59.3)	486	(71.4)
	Other	1040	(49.3)	483	(40.7)	195	(28.6)
Category of hospital, n (%)							
	Charity Hospital	814	(38.6)	587	(49.5)	377	(55.4)
	Private Hospital (non-philanthropic)	245	(11.6)	175	(14.8)	38	(5.6)
	Public Hospital	1050	(49.8)	424	(35.8)	266	(39.1)
Implant technique – mini-thoracotomy, n (%)		0		0		227	(38.0)

Among patients with information about underlying disease, in the ICD-alone group (n = 760), 36% were diagnosed with Chagas’ heart disease and 25.1% with ischemic heart disease (Table 2). In the CRT-D group (n = 310), 12.3% were diagnosed with Chagas’ heart disease (Table 2). The underlying cardiac diseases by age groups were described in Table 3. Cardiac causes accounted for 79% of deaths in both groups (Table 4), and Chagas’ heart disease accounted for 33% and 23% of cardiac deaths in the ICD-alone and CRT-D groups, respectively (p < 0.05). Of all deaths, there were only six unattended deaths in the ICD-alone group and one in the CRT-D group.

**Table 2:** **Underlying cardiac disease by type of ICD**

**Underlying cardiac disease, n (%)**	**Type of ICD**			
	**ICD**		**CRT-D**	
Cardiomyopathy	172	(22.6)	202	(65.2)
Chagas’ heart disease	274	(36.1)	38	(12.3)
Congenital heart disease	45	(5.9)	0	(0.0)
Ischemic heart disease	191	(25.1)	61	(19.7)
Other causes (myocarditis, valvular heart disease, hypertensive heart disease)	78	(10.26)	9	(2.9)
Total	760	(100)	310	(100)

**Table 3:** **Underlying cardiac disease by age group**

**Underlying cardiac disease, n (%)**	**Age group, n (%)**															
	**<10 years**		**10 to 19**		**20 to 29**		**30 to 39**		**40 to 49**		**50 to 59**		**60 to 69**		**70 years or more**	
Cardiomyopathy	1	(100)	6	(42.9)	12	(44.4)	22	(44.0)	50	(42.0)	93	(32.4)	111	(33.0)	78	(33.2)
Chagas’ heart disease	0	(0.0)	0	(0.0)	4	(14.8)	18	(36.0)	44	(37.0)	96	(33.4)	100	(29.8)	50	(21.3)
Congenital heart disease	0	(0.0)	4	(28.6)	4	(14.8)	0	(0.0)	8	(6.7)	6	(2.1)	13	(3.9)	10	(4.3)
Ischemic heart disease	0	(0.0)	2	(14.3)	4	(14.8)	6	(12.0)	10	(8.4)	65	(22.6)	89	(26.5)	76	(32.3)
Other causes (myocarditis, valvular heart disease, hypertensive heart disease)	0	(0.0)	2	(14.3)	3	(11.1)	4	(8.0)	7	(5.9)	27	(9.4)	23	(6.8)	21	(8.9)
Total	1	(100)	14	(100)	27	(100)	50	(100)	119	(100)	287	(100)	336	(100)	235	(100)

**Table 4:** **Causes of death by type of ICD**

	**Type of ICD**					
**Underlying cause of death**	**Single-chamber ICD**		**Dual-chamber ICD**		**CRT-D**	
	**(n = 2,109)**		**(n = 1,186)**		**(n = 681)**	
Chagas’ heart disease	167	(26.0%)	42	(26.8%)	36	(18.3%)
Cardiac diseases (other)	340	(53.0%)	82	(52.2%)	119	(60.4%)
Noncardiac vascular diseases	17	(2.6%)	5	(3.2%)	8	(4.1%)
Cancer	16	(2.5%)	2	(1.3%)	4	(2.0%)
Infection	12	(1.9%)	2	(1.3%)	0	(0.0%)
Other	90	(14.0%)	24	(15.3%)	30	(15.2%)
Total	642	(100%)	157	(100%)	197	(100%)

**Figure 1:**
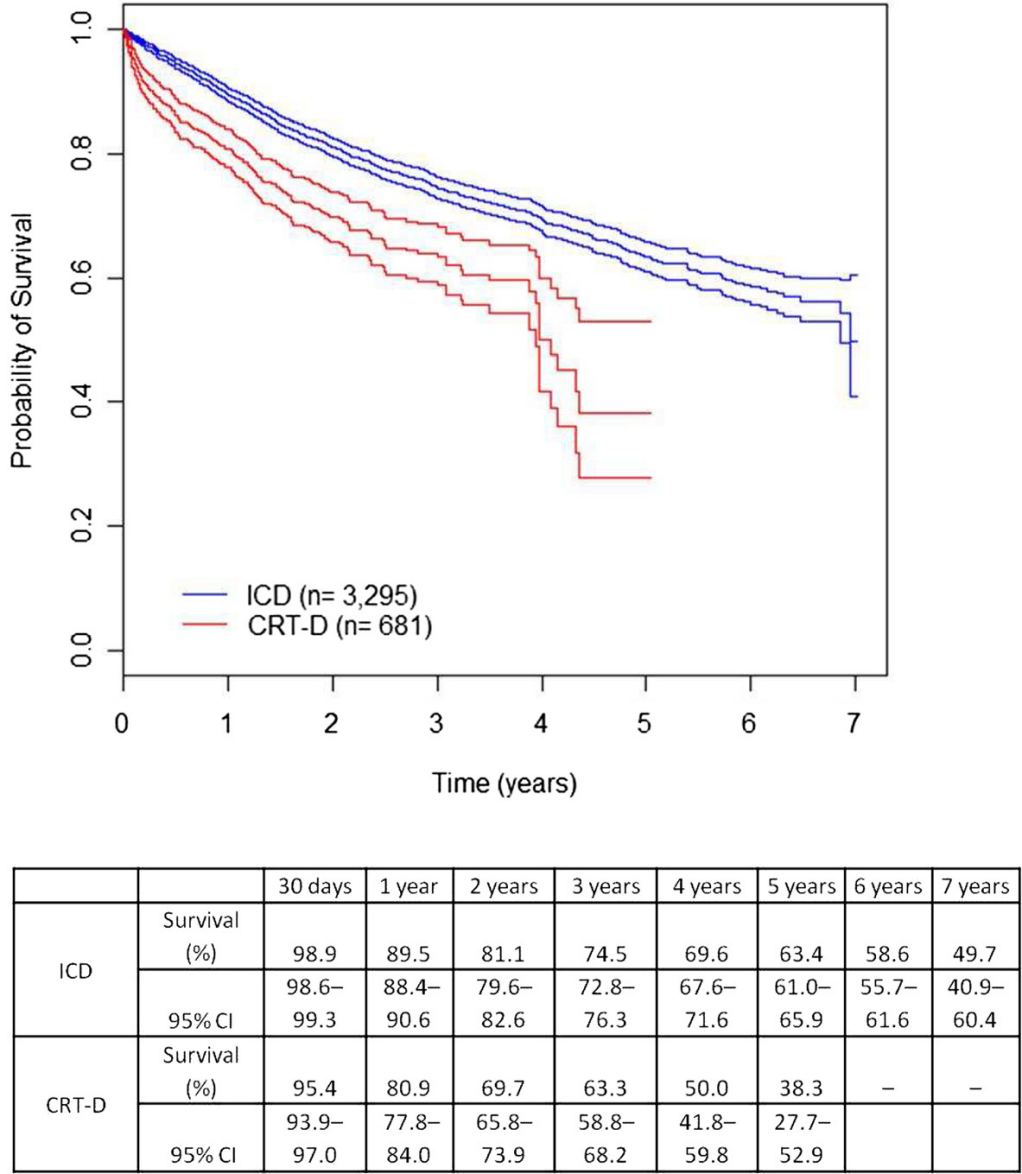
**Overall survival by therapy (ICD-alone or CRT-D).** Kaplan-Meier survival estimates were significantly different between the two groups (95% CI).

**Figure 2:**
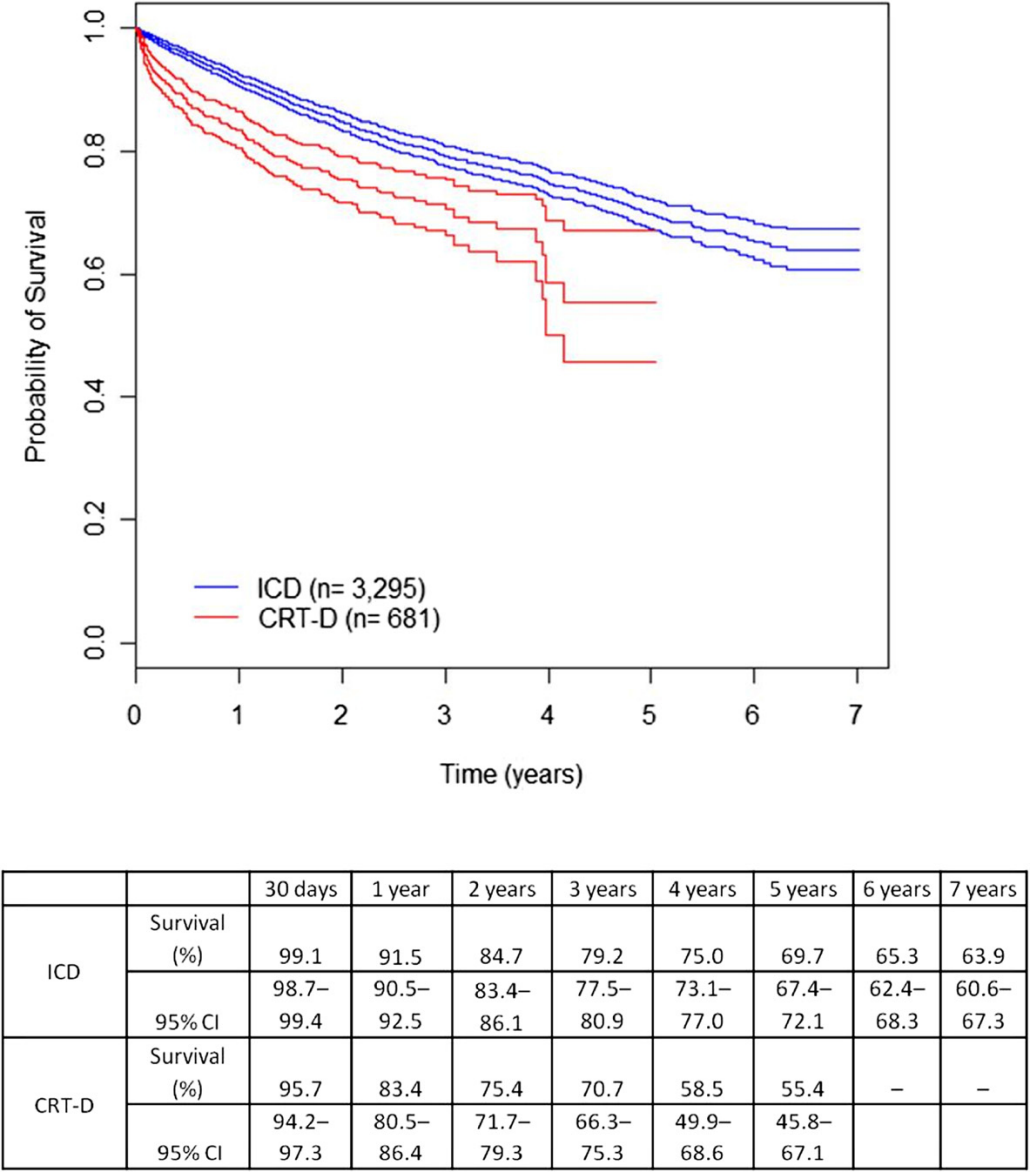
**Cardiac survival by therapy (ICD-alone or CRT-D).** Kaplan-Meier survival estimates were significantly different between the two groups (95% CI).

The mean length of hospital stay for device implantation was shorter in ICD-alone compared to CRT-D patients (5.8 vs. 7.7 days, p <0.001). The in-hospital case fatality was 0.3% in the ICD group and 2.9% in the CRT-D group (p <0.001). The in-hospital case fatality of CRT-D patients over 70 was 8.5%. Overall short-term, medium-term and long-term survival and short-, medium- and long-term cardiac survival (30 days, 1 year and 5 years, respectively) are presented in Figures 1 and 2, stratified by type of therapy. The differences found in survival times of the ICD and CRT-D groups were statistically significant for all periods studied, for both overall and cardiac survival (Figures 1 and 2). Patients in the CRT-D group showed poorer prognosis (Figure 1). A marked drop in survival was evident in the CRT-D group around the fourth year of observation (Figure 1), with a decrease in survival rates from 59.5% (95% CI 54.3–65.3) to 38.3% (95% CI 27.7–52.9) in only 5.5 months.

The analysis of overall survival of ICD patients showed that age was associated with the outcome (HR 1.03, 95% CI 1.03–1.04), with 3% increase in the risk of death per year. This association remained in the multivariate models for both overall and cardiac survival. There were no deaths in children in our study. In the ICD group, the 1-year survival was 93.8% (95% CI 92.2–95.5) in patients aged 10 to 49 years and 81.6% (95% CI 78.3–85.1) in those 70 years or more. No other variables studied were significantly associated with the outcome in either group.

**Figure 3:**
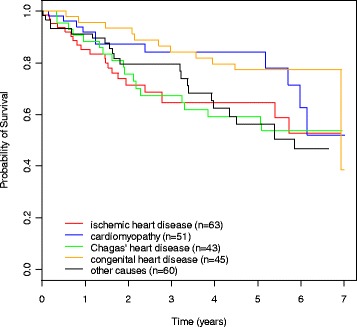
**Overall survival by underlying cardiac disease (ICD-alone group).** Kaplan-Meier survival estimates were not significantly different between groups (Peto’s test p = 0.05). These survival curves with underlying disease information drawn only from the hospital admission (AIH) forms.

Figure 3 shows survival curves with underlying disease information drawn from the AIH for the ICD-alone group. This Figure does not include the information on underlying disease drawn from the mortality database, because – as we used specific data on patients who died – survival would be artificially low. Kaplan-Meier survival estimates were not significantly different (Peto’s test, p = 0.84) between the two groups (with and without underlying disease at SIH), suggesting random loss of these information. The cardiac survival curves were very similar to these overall survival curves, showing the same pattern.

**Figure 4:**
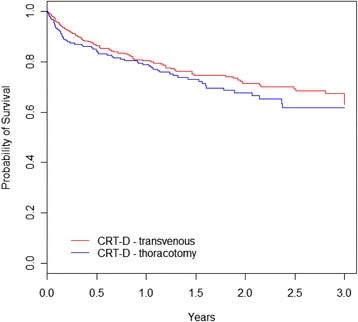
**Overall survival by implant technique.** Kaplan-Meier survival estimates were not significantly different between the two groups (Peto’s test p = 0.263). The group we denominated ‘thoracotomy’ comprises two subgroups with survival curves overlaid: patients with surgically-implanted left ventricle leads as the first approach and patients with transvenous implantation failure, who were subsequently converted to thoracotomy.

In the CRT-D group, it was used a transvenous endocardial technique in 62% patients, epicardial in 28% and endocardial requiring thoracotomy due to implant failure in 10%. The in-hospital case fatality was 5.3% among those undergoing epicardial lead placement, which is higher than 1.6% found among those undergoing transvenous implantation (p <0.05) considering that both groups had a similar median length of hospital stay (4 days). Those requiring thoracotomy due to implant failure accounted for 25.1% of all patients undergoing epicardial technique and 16.7% of deaths in this group. There were no significant differences in the medium-term and long-term prognosis according to implantation technique (Figure 4).

## Discussion

To the best of our knowledge, this is the first study to analyze survival in all patients undergoing ICD and CRT-D implantation within a National Health System. This design allowed to avoiding selection bias and increasing the generalizability of effectiveness results.

Overall 1-year, 2-year and 3-year survival rates in the ICD-alone group were similar to those reported in the AVID study (89.3%, 81.6% and 75.4%, respectively) [17]. In addition, the rate of cardiac deaths was similar to that reported in the same study [18]. One-year, 2-year, 3-year, 4-year, and 5-year survival rates in the CRT-D and ICD groups were similar to those described in a large case series in the US [7]. The overall 5-year survival in our study was similar to that reported in the RAFT Trial (65.4%) among ICD patients, but considerably lower than that reported in CRT-D patients [19]. This difference in long-term prognosis may be explained by the inclusion of patients with NYHA functional class II or III in this study. The one-year survival in the CRT-D group was lower than the 88% described in the COMPANION trial, even when the group undergoing transvenous implant was considered individually [2].

The 5-year survival found among ICD patients in our study was very similar to that observed in a study of patients with Chagas’ heart disease [10]. Although Chagas disease was the underlying cause in one-third of all patients who eventually died, we cannot infer that these patients had a poorer prognosis. Our survival curves by underlying disease suggests that etiology of heart disease (Chagas vs. ischemic heart disease) was not a prognostic factor. The ICD-LABOR study that also included patients with Chagas’ heart disease found similar results [20]. Barbosa et al. suggested that Chagas’ heart disease patients are more likely to have ventricular arrhythmias than patients with other cardiopathies, but on the other hand, they have higher rates of appropriate ICD therapy [21].

The in-hospital case fatality of 0.33% observed in the ICD group in our study was only slightly higher than the 0.2% reported in a systematic review of nonthoracotomy ICD trials [22] but considerably lower than that reported in other clinical data analyses [12,23,24]. Nevertheless, the 1.1% 30-day case fatality observed was identical to that reported in the AVID trial, but higher than the 0.6% found in a systematic review of nonthoracotomy ICD trials by Rees et al. [22]. The mean length of hospital stay among ICD patients was shorter than that reported in a large study of administrative databases in the US [12].

The in-hospital case fatality of 2.9% seen in the CRT-D group was higher than the 0.5% case fatality reported in a systematic review [25], 0.9% reported by Swindle et al. [23] and 1.1% reported in Medicare patients [24]. The COMPANION and MADIT-CRT trials also found much lower in-hospital case fatality (0.6% and 0.1%, respectively) than that found in the present study; however, both studies excluded patients with implantation with thoracotomy and the MADIT-CRT trial included only patients with NYHA Class I and II [22]. Even the in-hospital case fatality in the CRT-D sub-group undergoing transvenous implantation (1.6%) was higher than those reported in these studies. The 8.5% in-hospital case fatality in CRT-D patients older than 70 was much higher than that reported by Swindle et al. in elderly patients [23]. The 30-day mortality in those undergoing transvenous CRT-D implantation in our study (4.1%) was higher than the 1.8% observed in the COMPANION trial [2]. Even among younger patients, the mean length of stay in the CRT-D group was higher within SUS than that reported in Zhan et al. study [12]. The median length of hospital stay in our study was the same regardless of the implant technique.

Our study did not find any deaths in children undergoing ICD implantation. Other study corroborate the good prognosis in this age group [9]. Differences in survival times between ICD-alone or CRT-D patients were expected because the indication criteria for CRT-D implant included poor functional status (NYHA Class III or IV) and ventricular dyssynchrony.

The decrease in survival among CRT-D patients around the fourth year of follow-up may suggest the impact of the disease natural history or device-related problems. A study by Cleland and colleagues with patients with heart failure and dyssynchrony found among those treated with medical therapy alone a pattern of decline in survival mainly due to sudden death that is similar to that observed in our study [26]. However, this pattern of survival was not observed in Saxon and colleagues study that also assessed long-term outcomes [7]. One explanation for this pattern would be the effect of a factor affecting the long-term effectiveness of CRT-D therapy and its impact in the disease natural history. Horlbeck and colleagues showed that mean lifetime of CRT-D devices was 4 years [27]. Likewise, Biffi and colleagues demonstrated that median lifetime of CRT-D devices was approximately 4 years [28]. Thijssen and colleagues reported that mean battery lifetime of CRT-D devices was 4.7 years [29]. In an earlier study by Hauser and colleagues (1998–2005) only 4% of CRT-D pulse generators were operating normally within four years of the implant [30]. A recent study by Landolina and colleagues reported that at three years slightly more than 10% of patients underwent surgical revision for battery depletion and at four years this rate rose to about 50% [31]. This same study showed that patients undergoing replacement had double the risk of infection, which could also explain the mortality observed in our study. Only ongoing monitoring can establish whether this finding was exclusive to the initial cohort of patients undergoing CRT-D implantation (long follow-up) and to what extent it was impacted by the small number of patients at-risk in the fourth year of follow-up.

Dual-chamber devices accounted for 65% of all ICD implants In the last three years studied, a percentage that is similar to that reported in the US National ICD Registry (62% from 2006 to 2007) [8]. In this study only 40.4% of patients implanted with dual-chamber ICD devices met the indications for pacemaker therapy and the use of dual-chamber ICD devices was associated with increased in-hospital complications and in-hospital case fatality rates [8]. In our study we found no differences in in-hospital case fatality and short-term, medium-term and long-term survival rates among those implanted with single- and dual-chamber devices. Those undergoing dual-chamber ICD implantation were less likely to have supraventricular tachycardia, which contrasts with that reported by Dewland and colleagues [8], suggesting that this is not a common indication for atrial lead placement in Brazilian patients.

The present study showed that the proportion of CRT-D with surgically implanted left ventricle leads as first approach (28%) seems slightly greater than that reported in other studies (24.1% to 24.9%) [32,33]. The percentage of transvenous implantation failure in our study (13.7%) was also higher than that reported in other studies with CRT-D (5.9% to 11%) [32-34]. In patients undergoing CRT-D implantation, epicardial lead placement was found associated with increased in-hospital case fatality, as suggested in other studies with CRT [35] and CRT-D [32]. Despite increased in-hospital case fatality and 30-day case-fatality, we found similar medium-term and long-term survival rates for both implantation techniques suggesting similar effectiveness of these techniques in patients surviving the initial post-implantation period, which corroborates that reported by Miller and colleagues [35] with CRT. However, conflicting results have been reported. One study showed poorer short-term and long-term prognosis with the epicardial technique [32] while other studies found no significant differences in short-term or long-term results [33,36]. The results found in our study regarding the epicardial technique may be explained by the “learning curve” of surgical teams, quality of postoperative care and potentially suboptimal drug therapy prescribed to patients with advanced heart failure referred to surgery [37].

Our study has some limitations common to studies relying on administrative databases. The lack of information on patient variables such as NYHA functional class, left ventricle ejection fraction, and history of sudden cardiac death does not allow proper adjustment for the patients’ baseline risk. The proportion of underlying diseases may not reflect their actual distribution in the study population due to missing information on this variable in SIH database. Information on the underlying condition is often missing in hospital admission authorization forms because providers are required to fill out two different diagnosis fields including diagnostic codes to support the patient’s eligibility for device implantation (e.g., type of arrhythmia and heart failure). The sensitivity of the probabilistic record linkage method used to find deaths registries (90.6%) may have potentially missed some death records in the database. However, there was 100% specificity in identifying deaths records and probably random loss [16].

## Conclusion

Our study showed that the medium-term and long-term effectiveness of ICD therapy in Brazil appears to be similar to the efficacy found in clinical trials. However, there is an apparent slight excess of deaths within the first 30 days of implantation. Younger age at the time of implantation was a predictor of better prognosis in the ICD-alone group. In the CRT-D group, in-hospital case fatality and 30-day case fatality were higher than those reported in other studies. There was a marked drop in survival around the fourth year after implantation, and further investigation is necessary to determine its causes. In addition, the epicardial implantation technique was more frequently used in the CRT-D group than that reported in the literature and was found to be associated with poorer short-term prognosis. The study results suggest there is still room for reducing the proportion of surgical procedures within SUS, and more importantly, actions should be taken to reduce mortality associated with surgical CRT-D implantation and transvenous implantation of both ICDs and CRT-Ds.

## Abbreviations

ICD: Implantable cardioverter-defibrillator
CI: Confidence interval
CRT-D: Cardiac resynchronization therapy-defibrillator
EF: Ejection fraction
HR: Hazard ratio
NYHA: New York Heart Association
SIH: Brazilian Hospital Database
SIM: Brazilian National Mortality Database
SUS: Brazilian National Health System
AIH: Hospital admission authorization forms
